# Multifactorial Etiology of Adolescent Nicotine Addiction: A Review of the Neurobiology of Nicotine Addiction and Its Implications for Smoking Cessation Pharmacotherapy

**DOI:** 10.3389/fpubh.2021.664748

**Published:** 2021-07-05

**Authors:** Supriya D. Mahajan, Gregory G. Homish, Amanda Quisenberry

**Affiliations:** ^1^Department of Community Health and Health Behavior, School of Public Health, University at Buffalo, Buffalo, NY, United States; ^2^Department of Health Behavior, Roswell Park Comprehensive Cancer Center, Buffalo, NY, United States

**Keywords:** nicotine, neuroscience, microglia, etiology, addiction, adolescent

## Abstract

Nicotine is the primary pharmacologic component of tobacco, and its highly addictive nature is responsible for its widespread use and significant withdrawal effects that result in challenges to smoking cessation therapeutics. Nicotine addiction often begins in adolescence and this is at least partially attributed to the fact that adolescent brain is most susceptible to the neuro-inflammatory effects of nicotine. There is increasing evidence for the involvement of microglial cells, which are the brain's primary homeostatic sensor, in drug dependence and its associated behavioral manifestations particularly in the adolescent brain. A hallmark of neuro-inflammation is microglial activation and activation of microglia by nicotine during adolescent development, which may result in long-term addiction to nicotine. This non-systematic review examines multifactorial etiology of adolescent nicotine addiction, neurobiology of nicotine addiction and the potential mechanisms that underlie the effects of nicotine on inflammatory signaling in the microglia, understanding how nicotine affects the adolescent brain. We speculate, that modulating homeostatic balance in microglia, could have promising therapeutic potential in withdrawal, tolerance, and abstinence-related neural adaptations in nicotine addiction, in the adolescent brain. Further, we discuss nicotine addiction in the context of the sensitization-homeostasis model which provides a theoretical framework for addressing the potential role of microglial homeostasis in neural adaptations underlying nicotine abuse.

## Introduction

Nicotine addiction is the leading cause of preventable death and disease worldwide. Preclinical models and human studies have demonstrated that nicotine has cognitive-enhancing effects and these effects of nicotine may be an important factor in vulnerability to Tobacco Use Disorder (TUD) and may also contribute to difficulty in quitting smoking. The positive reinforcement effects of nicotine reflect nicotine's inherently rewarding effects that increase the probability of continued self-administration, and for both, the initiation and maintenance of tobacco use (1–3). Preclinical models typically used cell cultures or animal models that involve administration of nicotine to rodents. Preclinical models have consistently demonstrated that nicotine had both neuroprotective and anti-inflammatory effects depending on the nicotine dose administered and nicotine enhanced neurotrophic factors, increased cognition and impulsivity and developed neurotoxicity in the developing brain (2–4). Clinical studies evaluated the neurotoxic effects of tobacco smoking on the brain, and also evaluated the cognitive and behavioral assessments, as well as neuroimaging measures in the human brain, and have established that tobacco smoking decreases brain volume, increases neuro-inflammation and oxidative stress but enhances cognition and neural efficiency (4).

The National Institute of Drug Abuse (NIDA) reports that tobacco use is established primarily during adolescence, and evidence suggests that around 50% of those who start smoking in the adolescent years continue to smoke for 15–20 years (5). A National Youth Tobacco Survey (NYTS) found that 41.9% adolescents reported strong cravings for tobacco- a classic symptom of nicotine dependence (6). In adolescents, even infrequent smoking can result in an increased risk of dependence. In adolescents, monthly smoking can increase the likelihood of developing nicotine dependence by 10-fold as compared to adult smokers (7–10). The urge to smoke occurs early on after initiation, which drives the increase in frequency of use, exacerbating into nicotine dependence and a more rapid progression to addiction and a neurophysiologic dependence on nicotine (9). The risk of nicotine dependence in adolescents is associated with intensity of recent cigarette consumption, a slower nicotine metabolism and depression (11). The CDC warns that if cigarette smoking continues at the current rate among youth, 5.6 million of Americans younger than 18 will die early from a smoking-related illness (12).

Adolescence is a period of transition characterized by significant hormonal, psychosocial, and neural changes (13). This period is associated with development of social, emotional, and cognitive skills and also increased vulnerability to stress and risk-taking behaviors (14–16). The adolescent brain is undergoing maturation and is particularly vulnerable to the harmful effects of drugs of abuse, including tobacco and nicotine containing products. Nicotine binds to nicotinic acetylcholine receptors (nAChRs). nAChRs are widely distributed throughout the human brain and are critical in neurotransmitter release, brain maturation, reward processing, and cognition (17). Nicotine exposure during adolescence, disrupts the normal development, and expression of neuronal nAChRs, ultimately altering the function and pharmacology of the receptor subunits and changing the release of reward-related neurotransmitters (18).

E-cigarettes have emerged as the most common mode of nicotine delivery among youth across the U.S and its use is most prevalent among adolescents' and by vaping nicotine products, adolescents' do not have an awareness and understanding of nicotine and its presence within E-cigarettes products (19, 20). In adults, e-cigarettes are a potential cessation aid, while among adolescents who have never before smoked, e-cigarette use is associated with initiation or escalation of cigarette smoking (21, 22).

Smoking prevalence is a function of multiple parameters, such as initiation, cessation and relapse. Prevalence of adult smoking and cessation are both correlated with levels of childhood smoking intensity (23, 24). Adolescent smokers were the most likely to relapse and are more vulnerable to peer pressure which makes them more susceptible to smoking relapse after cessation (25). Adolescent smokers may underestimate the health consequences of smoking and therefore limit their determination to quit (26). A recent study that examined reuptake and relapse to tobacco use across a variety of tobacco products such as cigarettes, electronic nicotine delivery systems, cigars, hookah, and smokeless tobacco showed that for all the tobacco products reuptake occurred in 7.8% of adult previous users and 30.3% of adolescent previous users (27). These data affirm that preventive strategies should be designed early, so as to reduce, delay, or eliminate any youth access to cigarettes.

First-line pharmacologic therapies for smoking cessation includes nicotine replacement therapy (NRT), varenicline, and bupropion, however, the choice of therapy is based largely on patient preference. For those smokers willing to quit, a combination of behavioral support and pharmacologic therapy is the most effective in smoking cessation (28, 29). FDA has not approved cessation medications for adolescents, and NRT cannot be purchased over-the-counter by persons younger than 18 years of age (30, 31), but cessation medications can be prescribed for and used by adolescents under the supervision of a physician. A systematic meta-analysis study detected no significant efficacy of pharmacological therapy in adolescents, therefore, no definitive recommendations for pharmacotherapy for smoking cessation in adolescents could be made (32, 33). The tobacco cessation 5-A method (ask, advise, assess, assist, and arrange) that is used by adults, should be offered by the physician to all adolescents who smoke after assessment of the level of tobacco dependence in the adolescent using the Fagerström test for nicotine dependence (34). Therapies for adolescents should include counseling, nicotine replacement therapy, psychoactive medication (e.g., bupropion), and combination therapy (35).

Currently available cessation strategies include community interventions such as educational programs; anti-tobacco counter-advertising at the local, state, and national levels and curtailing access to tobacco via smoking bans at home and school and increased tobacco prices in combination with pharmacotherapy, all of which may be effective in decreasing tobacco use in adolescents. Novel smoking cessation experimental interventions using text messaging (36) peer mentoring (37) and digital or virtual self-help interventions (38) for adolescents may be more effective, however data supporting the effectiveness of such interventions at the current time are limited, however experts suggest that these novel strategies when used in combination with counseling and pharmacotherapy may be very effective (39).

Effects of nicotine are highly dependent on when exposure to the brain occurs and contributes to specific neural vulnerabilities at each brain developmental phase. Several studies have shown that prenatal, early postnatal, and adolescent brain maturation is physiologically regulated by acetylcholine (ACh) via activation of nicotinic acetylcholine receptors (nAChRs), and that nicotine exposure results in significant long-term deficits in the developing brain by interfering with the cholinergic regulatory processes (40–42). The dopaminergic system is dynamically changing during adolescence and stimulation by nicotine alters maturation of the mesocorticolimbic system via the nAChRs on dopaminergic neurons and microglia (43).

Given the susceptibility of the developing brain to nicotine as outlined above, preventing tobacco product use among youth is critical to ending the tobacco epidemic in the United States. Tobacco smoking continues to be the leading cause of preventable morbidity and mortality globally (44) which underscores the need for better therapeutics for nicotine dependence. In order to develop more effective therapeutic interventions, it is essential not only to understand the pathophysiology of addiction but also examine the adolescent neurobiology and the genetic predisposition that underlies the etiology of adolescent nicotine addiction.

## Method

We conducted a non-systematic literature review to examine in depth the multifactorial etiology of adolescent nicotine addiction. The review is largely based on a selection of current, high-quality articles in the field of neuroscience and epidemiology relevant to nicotine addiction with the goal of examining a potential relevant model, such as the sensitization-homeostasis model, which not only explains the development of nicotine addiction in adolescents, but is also strongly supported by scientific literature.

### Multifactorial Etiology of Adolescent Nicotine Addiction

#### Parental and Peer Influence

Epidemiological and clinical data have shown that exposure to tobacco or nicotine can lead to subsequent abuse of nicotine and other recreational drugs in adolescents, and this phenomenon is described as the gateway hypothesis (45). Parents can affect the health of their children through genetic factors, physical and mental health, health behaviors and socioeconomic status (11).

Nicotine dependence, depression, and parental socioeconomic factors, contribute significantly to poor health in early adulthood and adolescence (46).

Parental smoking and nicotine dependence directly increases child onset of smoking, daily smoking and nicotine addiction (47). Peer influence on the etiology and maintenance of smoking is enormous and predicts initiation, smoking persistence and dependence, and is also a mediator or progression to substance abuse (48). Although adolescent behavioral and personality characteristics may be associated with initiation, and continued use of cigarettes, individual genetic differences in initial sensitivity to nicotine may constitute a critical element in adolescent susceptibility to nicotine dependence (49).

#### Genetic Influence on Nicotine Dependence

Genetic Predisposition confers liability to nicotine dependence and variation in individual genes have been associated with nicotine dependence. The evidence for a significant role of genetic factors on nicotine dependence is substantial. Both linkage studies and genome wide association studies (GWAS) have identified candidate genes/genomic regions associated with nicotine dependence (50–53). Measured genetic variation are also associated with nicotine dependence treatment efficacy (54).

**Figure 1:**
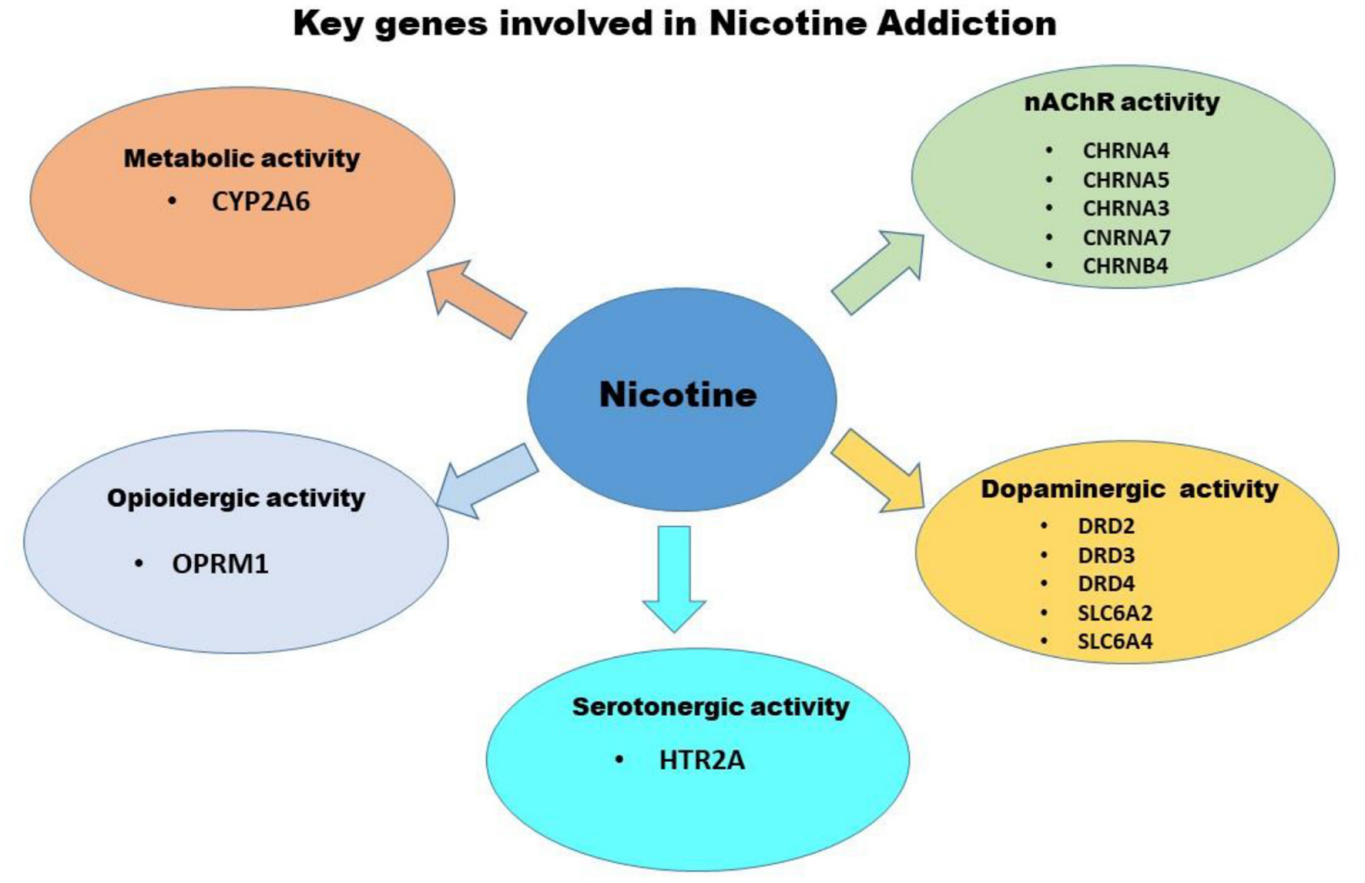
Schematic of Genetic factors that significantly influence both smoking initiation and persistence. Highlighted are genes associated with differences in nicotine's metabolic capacity and nicotine effects on central nervous system neurotransmitter functionality, specifically the those that modulate direct and indirect effects on nAChR, dopaminergic and opioidergic activity.

Genetic factors significantly influence both smoking initiation and persistence, a schematic of these influences are presented in Figure 1, these include genes associated with differences in nicotine's metabolic capacity and nicotine effects on central nervous system neurotransmitter functionality, specifically the dose that modulate direct and indirect effects on nAChR, dopaminergic and opioidergic activity, respectively. The candidate genes that play a key role in nicotine addiction include those associated with the dopaminergic neurotransmitter system (e.g., DRD2, DRD3, DRD4), cellular transport system (e.g., SLC1A2, SLC6A4), serotonergic neurotransmitter system (e.g., HTR2A), nicotinic neurotransmitter system (e.g., CHRNA4, CHRNA5, CHRNA3, CHRNA7, CHRNB4), opioidergic activity (e.g., OPRM1), and nicotine metabolism (e.g., CYP2A6) (55).

nAChRs are primary targets of nicotine, nicotine exerts direct and indirect effects on other receptor systems (e.g., opioid, serotonergic, glutamatergic) that also mediate nicotine-induced behavioral and neural changes in humans. Variation in the genes that code for the drug receptor proteins or that code for metabolic and catabolic enzymes that influence neurotransmitter levels, also represent the candidate genes for nicotine dependence and treatment.

The CYP2A6 genotype confers a slow nicotine metabolism increasing the risk of nicotine dependence (56). CYP2A6, is a genetically variable hepatic enzyme that is responsible for the majority of the metabolic inactivation of nicotine to cotinine. This enzyme mediates over 90% of the conversion of nicotine to cotinine, which is a major route of elimination of nicotine and therefore CYP2A6 activity is an important indicator of nicotine metabolism. A slow rate of nicotine conversion into cotinine results in a prolonged presence of higher nicotine concentrations in the bloodstream, thus increasing the exposure of nicotinic acetylcholine receptors in the brain to nicotine. Variant alleles of the CYP2A6 gene are associated with slower nicotine metabolism (57).

### Neurobiology of Nicotine Dependence

Nicotine from a smoked cigarette reaches the brain in as little as 7 s after inhalation (58). Inhalation of cigarette smoke results in nicotine quickly crosses the blood brain barrier and binding to nicotinic acetylcholine receptors (nAChRs) in the brain (59). Activation of nAChRs stimulates the mesocorticolimbic dopamine system which is the reward pathway thus producing the primary reinforcing effects of nicotine (60). Stimulation of dopamine neurons in the ventral tegmental area (VTA) by nicotine via high affinity α4β2 nAChRs causes increased firing in terminal dopaminergic fields, such as the nucleus accumbens (NAc), amygdala, and the prefrontal cortex (PFC) (61).

Exposure to nicotine in conjunction with environmental cues, causes lasting changes in dopaminergic function, which contribute to maintenance of smoking and the experience of withdrawal symptoms upon cessation (62–64). Disruption of dopaminergic activity via pharmacological blockade of dopamine receptors and disruption of nAChRs leads to decreased nicotine-induced reinforcement, suggesting a mediating role of these receptors in the reinforcing properties of nicotine (65).

### Vulnerability to Nicotine in Adolescence

Nicotine is a psychoactive and addictive substance that directly acts on brain areas involved in emotional and cognitive processing. Preclinical and clinical data suggests that although sociocultural influences significantly affect smoking adolescence, adolescent sensitivity to nicotine has strong neurobiological underpinnings (66).

Adolescence is a sensitive period for maturation of brain circuits that regulate cognition and emotion, with resulting vulnerability to the effects of nicotine and tobacco (67, 68). Adolescence is defined as a transitional period from childhood to adulthood that is conservatively estimated to last from 12 to 18 years of age in humans, however the boundaries of this period and what it encompasses is debatable and can vary widely depending on gender, socioeconomic status, and nutritional state (13).

Adolescence is marked by major physical changes in the body, however the hallmark of this period is a major reorganization of forebrain circuitry (13). During adolescence, the brain is sensitive to novel experiences with major experience-dependent plasticity occurring in the prefrontal cortex (PFC) region of the brain that is responsible for executive control and decision-making (69). Thus, dynamic structural and functional reorganization of the brain occurs during adolescence.

### Structural Changes in the Adolescent Brain

The structural changes in the adolescent brain include prolonged reorganization of gray matter, white matter, and associated neurochemical systems. During adolescence there is a significant decrease in the gray matter volume and density in the prefrontal cortex, parietal cortex and basal ganglia, which are critical brain regions for executive function, sensory processing, and motivation (70–72). On the other hand, there are corresponding increases in white matter, which reflect increased myelination and axonal diameter, and result in increased efficiency of impulse transduction (73). These changes in gray and white matter are not homogeneous and this imbalanced maturation of subcortical emotional and reward-focused systems as well as cortical executive and impulse control systems are believed to underlie the increased risk-taking behavior in adolescence (74, 75). These significant structural changes in the brain during adolescence, are accompanied by neurochemical changes which are paralleled by increases in functional connectivity, all of which synchronously play a significant role in the development of executive function and cognitive control attributed, to the maturation of the dopamine system (76, 77).

A functional MRI study examined the effects of nicotine dependence and tobacco consumption on brain structural changes in young adolescents and found that nicotine dependence was associated with distinct atrophy patterns on the brain volume (78). Mild nicotine dependence displayed more structural brain alternations than the heavy nicotine dependence and is attributed to the intensified neuroplasticity, a neural adaptation the adolescent brain undergoes against brain atrophy (79).

Studies have shown that micro-circuitry of the PFC and the NAc show developmental differences in dopamine function indicating that that cognitive processing within these regions is profoundly different in adolescence as compared to adulthood (66). Thus, rapidly maturing dopamine systems may be especially sensitive to disruption by environmental influences during adolescence, with long-term consequences on addiction behavior.

Smoking during adolescence increases the risk of developing psychiatric disorders and cognitive impairment in later life (80, 81). In addition, adolescent smokers suffer from attention deficits, which aggravate with the years of smoking (82–84). Recent studies in rodents reveal the molecular changes induced by adolescent nicotine exposure that alter the functioning of synapses in the PFC and that underlie the lasting effects on cognitive function (85). The PFC, is the brain area responsible for executive functions and attention performance, is one of the last brain areas to mature and is still developing during adolescence which makes the adolescent brain vulnerable to imbalance and therefore more susceptible to the influence of psychoactive substances such as nicotine (86). In prefrontal networks nicotine modulates information processing on multiple levels by activating and desensitizing nicotine receptors on different cell types and in this way affects cognition (87).

### Nicotine Induced Neurobiological Changes in Adolescents

Comparison of smoking behavior of adolescents with that of adult's point to an enhanced sensitivity of the adolescent brain to addictive properties of nicotine. Adolescents report symptoms of dependence even at low levels of cigarette consumption (88, 89). Adolescents are uniquely sensitive to nicotine and therefore, understanding the distinct effects of nicotine use on the adolescent brain is critical to treating and preventing nicotine addiction. Nicotine interferes with adolescent brain maturation and causes persistent changes in neuronal signaling (41, 90). Nicotine exposure in adolescence modulates cortico-limbic processing and alters synaptic pruning patterns in reward-encoding brain regions (66, 91). Nicotine exposure may lead to higher levels of dependence by exerting neurotoxic effects in the prefrontal cortex (PFC) interfering with adolescent cognitive development, executive functioning, and inhibitory control (92). These effects are particularly evident under stressful or emotionally intense states and are most pronounced when smoking begins during early adolescence (93, 94). Neuronal nAChRs are central regulators of neurophysiology and signaling in addiction pathways and are widely distributed in neuroanatomical regions implicated in nicotine addiction (17). Adolescent nicotinic receptors in different neuroanatomical regions display significantly increased functionality compared with adult receptors (66, 95, 96). These data suggest that the underlying receptor mechanisms of nicotine tolerance differs between adults and adolescents, therefore the effectiveness of smoking cessation therapies differs between these group.

Adolescents also exhibit greater behavioral sensitivity and susceptibility to other drugs of abuse after nicotine exposure, contrary to that adults exposed to nicotine do not show enhanced behavioral sensitivity or susceptibility to other drugs of abuse (66, 97–99). The increased tolerance to nicotine in adolescents may contribute to an enhanced vulnerability, further the increase in adolescent nAChR functionality compared with adults may contribute to the shift in nicotine dependence (10, 41). Dopamine plays a large role in the rewarding effects of nicotine (66, 100). Since the dopaminergic system is still undergoing development during adolescence, nicotine-stimulated dopamine release is significantly higher during the early adolescent period (101).

In adults' dopamine release is attenuated during withdrawal, thus adolescents do not experience this same decrease in dopamine as adults and thus exhibit lower withdrawal symptoms and aversive effects (60, 102).

Nicotine withdrawal symptoms in adolescent smokers exhibit signs and symptoms that are characteristically associated with nicotine deprivation in adult smokers (103, 104). However, clinical studies suggests that the time course of withdrawal symptoms may be different for adolescents who are trying to achieve and maintain long-term abstinence and in those who have varying levels of nicotine dependence (10, 99).

### Microglia and Their Role in CNS Pathophysiology in the Context of Nicotine Addiction

Microglia are highly specialized resident immune cells of the brain and play a vital role in surveillance of the brain microenvironment, which enables them to detect and respond to perturbations by altering their own morphology based on the type of insult (105, 106). Recent studies have shown that microglia are critical mediators of anxiety-like behaviors in mice during nicotine withdrawal (107) and while microglia mediate both inflammatory responses in the brain and brain plasticity, little is known regarding their role in nicotine dependence and changes in microglial phenotypes in response to nicotine.

Adolescents are more to susceptible to microglial activation by nicotine as compared to adults which results in long term effects in terms of nicotine induced neuropathology and addiction (101, 108). Important structural and functional changes in synaptic plasticity and neural connectivity occur in different brain regions in adolescence (72, 74, 109). Most drugs of abuse activate microglia leading to a pro-inflammatory state which then alters neuro-circuits associated with reward and drug dependence (110–112).

Microglial activation phenotypes are described as (1) classic activation (M1 phenotype), (2) alternative activation (M2a phenotype), (3) alternative type II activation (M2b phenotype), and (4) acquired deactivation (M2c phenotype) (113, 114). M1 microglia are capable of producing reactive oxygen species (ROS) and produce cytokines such as tumor necrosis factor-α (TNF-α), IL-1β, IL-6, and IL-12, thereby mediating inflammatory tissue damage (115). The M1 phenotype is commonly referred to as neurotoxic (116, 117). M1 microglia regulate synaptic pruning (118) and exhibit limited phagocytic activity (119). M2a microglia exhibit significant phagocytic activity and respond to IL-4 and IL-13 stimulation by producing an insulin-like growth factor-1, anti-inflammatory cytokines such as IL-10; and to express G-CSF, GM-CSF, and CD209 (120–122). These microglia can stimulate tissue regeneration and can eliminate cellular debris. M2b microglia show increased IL-12, IL-10, and HLA-DR expression. M2b microglia also have significant phagocytic activity and an increased expression of CD32 and CD64. M2c also known as acquired deactivation phenotype is acquired as a result of stimulation with the anti-inflammatory cytokine IL-10 or glucocorticoids, shows increased expression of transforming growth factor (TGF), sphingosine kinase (SPHK1), and CD163 (123). The polarization of microglia toward the M2 phenotype occurs to resolve inflammation and degeneration as a whole; thus, this phenotype is characterized as neuroprotective (113, 114, 124, 125).

### Immunomodulatory Effects of Nicotine on Microglia

Nicotine induces both immunosuppressive and immuno-stimulatory effects in the CNS (126, 127). The translocator protein (TSPO) is used as a neuro-inflammatory marker as its expression is upregulated in reactive glial cells during CNS pathologies. However, it remains unclear in which microglial phenotypes TSPO levels are upregulated, as microglia can display a plethora of activation states that can be protective or detrimental to the brain. TSPO expression was selectively increased in M1 microglia but not M2 microglia. TSPO imaging reveals microgliosis in non-neurodegenerative brain pathologies, and this is perhaps reflected in the observation that cigarette smokers have decreased levels of TSPO suggesting that neuroprotective properties of nicotine and the anti-inflammatory responses of nicotine may be responsible for the decreased incidence in neurological diseases in smokers (128). Nicotine induced increases in brain inflammatory markers which are not only dose-dependent, but are also related to smoking intensity and time since smoking cessation (126). Neuroimaging studies, show gray-matter abnormalities throughout the brain, in smokers compared to non-smokers, and these are attributed to an upregulation of nicotinic acetylcholine receptors (nAChR) in the prefrontal cortex and the consequent differences in functional connectivity in the prefrontal cortex region in smokers compared to non-smokers (128, 129). Additional studies are needed to examine nicotine induced inflammatory responses and TSPO binding in human smokers during acute nicotine withdrawal in order to evaluate the therapeutic potential of microglial modulators as smoking cessation aids.

#### Studies in Adult Mice Models That Highlight Nicotine Induced Microglial Activation and Consequent Inflammatory Response

Adeluyi et al. showed that chronic nicotine treatment and nicotine withdrawal in adult mice both alter microglial morphology, however both conditions trigger different inflammatory responses in the brain NAc region (107). Chronic nicotine does not elicit a pro-inflammatory response in the NAc, but does induce microglial activation, while nicotine withdrawal does induce a pro-inflammatory response that involves the interaction between Nox-2, ROS, and TNFα in adult mice (107, 130). The NADPH oxidase (Nox) system is a major source of intracellular ROS production in the adult brain and the nicotine withdrawal induced activation of the Nox isoform-Nox-2 expression in microglia, which is believed to be the primary mechanism that results in increased ROS generation and pro-inflammatory response to nicotine withdrawal (131, 132).

### Role of Microglia in Nicotine-Induced Synaptic Plasticity

In addition to their immune function, microglia are also involved in synaptic function and plasticity; therefore, we speculate that, their alterations within the NAc, which is a critical brain circuit for addictive processes, may enhance the development of aberrant synaptic connections and plasticity underlying nicotine dependency (111). Synaptic cues specific to the NAc during exposure to chronic nicotine or withdrawal from chronic nicotine distinctly influence the phenotype of its resident microglia. Nicotine induced neuro-plastic changes that contribute to nicotine addiction are triggered with initial exposure to nicotine and cause significant changes in brain physiology, structure and function, and changes in behavioral responses. Microglia play a critical role in synaptic remodeling and plasticity that underlies drug addiction (133, 134). Nicotine can directly modulate microglial morphology and function via interaction with nicotinic acetylcholine receptors (nAChRs) on microglia (135, 136). α7-nAChR is the only nAChR subtype expressed by microglia (137–142).

### Role of Microglia in Nicotine-Induced Inflammation

Activated microglia produce and release a variety of pro-inflammatory cytokines and augmenting the production of free radicals (143). Microglial cells express innate immune receptors, Toll like Receptors (TLRs) and cytoplasmic NOD-like immune receptors (NLRs) (144, 145), which react not only to pathogens (PAMPs, pathogen associated molecular patterns), but also to stress conditions, and to cell damage (DAMPS or damage-associated molecular patterns) (146). Activation of TLRs triggers signaling pathways, such as the activation of transcription factor NF-κB, which produces cytokines and inflammatory mediators (146). Several studies demonstrate the participation of these receptors in neuroinflammation and associated neuropathology is induced by nicotine abuse, particularly in adolescence (147).

### Variable Effects of Nicotine on Adult and Adolescent Microglia

#### Morphological Differences

Significant morphological differences exist between adult microglia and adolescent microglia, adult microglia were larger and have more complex morphology than adolescent microglia.

#### Differences in Transcriptional Profiles

The transcriptional profile associated with immune activation is significantly different in adolescent microglia as compared to adult microglia (148). Nicotine treatment showed age-dependent effects on microglial marker Iba1 expression in the NAc and BLA which are actively maturing brain region during adolescence responsible for reward (66). Microglia express the receptor CX3CR1, which mediates developmental synaptic pruning through the neuronal ligand CX3CL1 (111).

#### Differences in Microglial Activation

Nicotine decreased overall expression of genes associated with microglial activation and nicotine alters the expression of these transcripts in an age-dependent manner which suggests that microglia are not fully mature by adolescence (101). A recent study showed that microglia are essential regulators of nicotine induced increases in cocaine seeking behavior (101) in adolescent microglia. Nicotine-induces microglial activation in the brain regions such as NAc, basolateral amygdala (BLA) which are responsible for reward (41, 66). The nicotine induced changes to microglial activation is mediated via the NAc localized D2 receptors and CX3CL1 signaling cascade suggesting that nicotine can induces significant changes to adolescent brain and behavior, and that microglial activation is a critical to this regulation (149). CX3CL1 not only mediates nicotine-induced increase in microglial activation, but increases the neuronal-microglial communication pathway via the CX3CL1-CX3CR1 interaction, after adolescent-nicotine exposure (149, 150).

Adult microglia treated with nicotine did not show either microglial proliferation nor activation demonstrating a neuroprotective effect of nicotine on adult microglia (151), however in the adolescent brain, nicotine treatment increased microglial activation (108) resulting in enhanced secretion of inflammatory cytokines, metabolic dysfunction, ineffective phagocytosis of proteins and neuronal debris, and alterations in neurochemical transmission producing long-term changes in limbic function (41, 152). The adolescence period is therefore a particularly vulnerable period during which, nicotine withdrawal induces microglial morphological changes in the nucleus accumbens (NAc) promoting microglial activation via Nox2-mediated increases in ROS.

#### Differences in Pro-inflammatory Response

**Figure 2:**
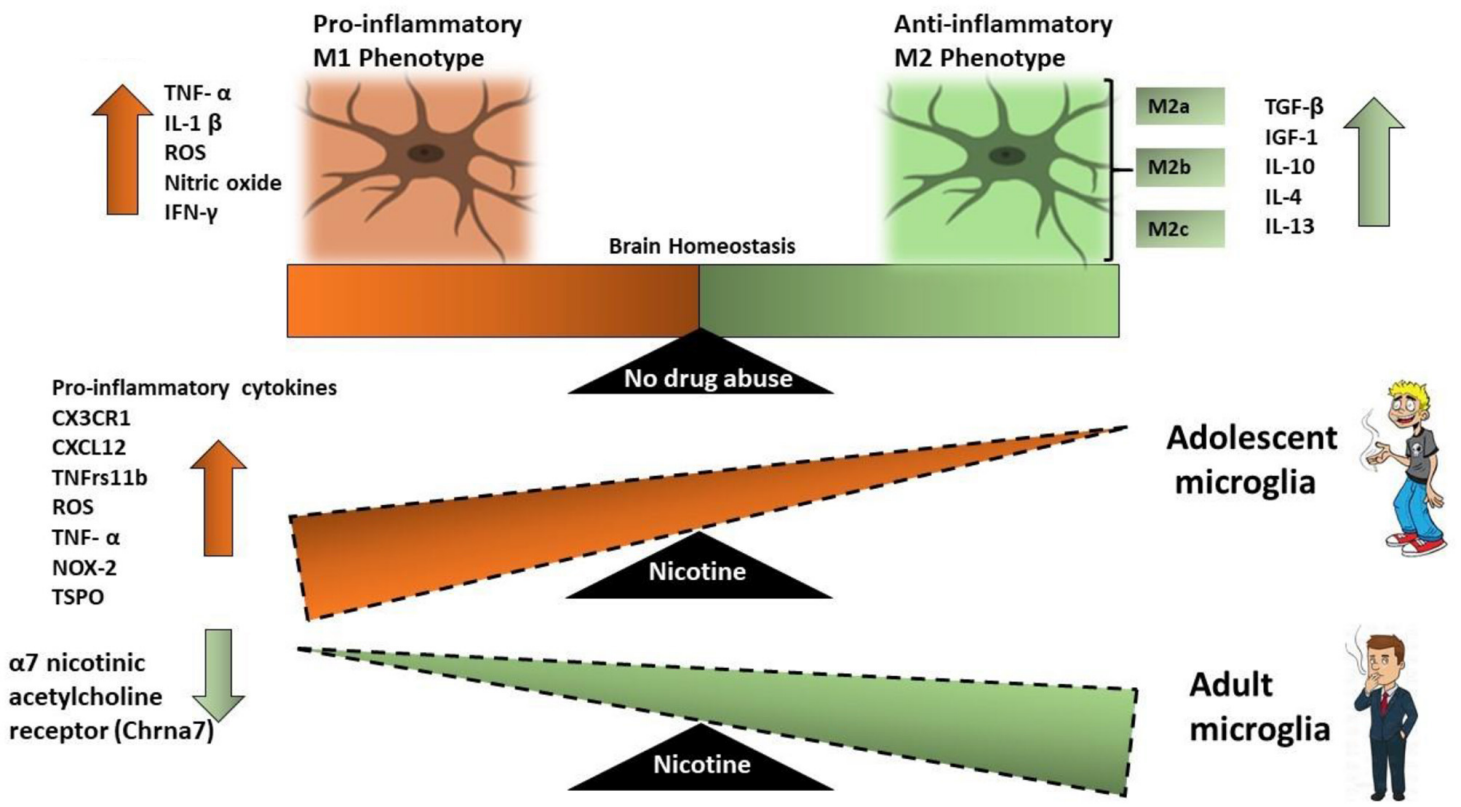
Schematic that illustrates the effect of nicotine on microglial activation in adult microglia vs. adolescent microglia. M1 microglia represent a neurotoxic environment with increased levels of pro-inflammatory cytokines while M2 microglia are neuroprotective. Adolescent-nicotine exposed microglia show an increased reactive M1 activation and a pro-inflammatory response. Increased expression of pro-inflammatory cytokines CX3CR1, CXCL12, TNFrs11β, ROS, NOX-2, TNF-α, and TSPO are reported in nicotine exposed adolescent microglia as compared to nicotine exposed adult microglia.

Once activated microglia release pro-inflammatory cytokines TNFα and IL-1β, which correlate with increased withdrawal behaviors (107, 153). The increase in the pro-inflammatory cytokines occurs in both adolescents as well as adults, however, the increase in inflammatory cytokines in adolescents is significantly higher than that in adults (101, 154) (Figure 2).

### Modulation of Microglial Activation—A Potential Therapeutic Approach for Cessation

Targeting the microglial potassium (KATP) channels has been shown to be effective in controlling inflammatory microglia activation, avoiding its toxic phenotype though a mitochondria-dependent mechanism (155). Such a strategy of modulating microglial activation and consequent neuroinflammation may be a novel therapeutic approach for treatment of nicotine withdrawal symptoms.

Nicotine withdrawal is associated with cognitive deficits including attention and episodic memory impairments. The presence of cognitive deficits correlated with microglial activation and the increased expression of neuro-inflammatory cytokines such as IL1β, TNFα and IFNγ, in the hippocampus and the prefrontal cortex regions of the brain (153). A non-steroidal anti-inflammatory drug (NSAID) such as indomethacin can prevent cognitive deficits and microglial activation during withdrawal, which suggests the potential use of anti-inflammatory agents to improve cognitive function during nicotine withdrawal (153).

The role of microglia in response to nicotine is further consolidated by experiments that show that microglial depletion reversed the microglial- related Nox2 and associated aberrant ROS production and also decreased anxiety-like behavior that is typical response to nicotine withdrawal (156).

Research investigating the role of microglia in nicotine dependence is limited and still novel, however, has potential implications in the development of more potent therapeutics to treat nicotine dependence and withdrawal.

### Future Pharmacotherapeutic Approaches to Treat Nicotine Addiction

Both the neurochemical and functional changes observed in adolescent brain regions are associated with dopamine modulation and the cerebral reward system, which are influenced by specific genes, suggesting that a genetic predisposition of the neural mechanisms is involved in the acquisition of dependence in nicotine addiction.

#### Use of Pharmacogenetics in Nicotine Addiction Treatment

Identification of genes involved in the inheritance of specific smoking phenotypes may strengthen the selection of treatment options tailored to individual genotype (157). Although evidence for associations of CYP2A6 with smoking behavior and for the nicotine-metabolite ratio as a predictor of relapse are promising, cost effectiveness of implementing pharmacogenomics therapy would depend on the distribution of the relevant genetic polymorphisms in all smoking individuals (158). Pharmacogenomics and nicotine dependence is still an emerging science.

#### Microglia as Therapeutic Target for the Treatment of Nicotine Addiction

We speculate that neurodevelopmental changes may be modulated by pharmacotherapy targeted to activate change in microglial phenotype which may promote brain homeostasis and a neuro-adaptation that favors decreased dependence on nicotine thus microglia are a promising therapeutic target that need to be explored. Currently, data on role of microglial activation in nicotine cravings, withdrawal and tolerance is limited. We believe that the sensitization-homeostasis model (159–161), which highlights the concept that “nicotine's dependence liability derives from its ability to stimulate neural pathways responsible for the suppression of craving and due to sensitization,” will provide a theoretical framework for addressing the potential role of microglial homeostasis in withdrawal, tolerance and abstinence-related neural adaptations in nicotine addiction in both adults and adolescents. The sensitization-homeostasis model is unique in its extensive integration of clinical observations and basic science and its attribution of dependence to craving suppression and suggests that separate homeostatic mechanisms are responsible for abstinence, withdrawal, and tolerance (162).

## Conclusion

Studies show that behavioral treatments particularly in adolescents are effective, whereas pharmacotherapies have only marginal success (28, 29, 32, 33). The side effect profiles for nicotine replacement therapy, bupropion, and varenicline in adolescents are similar to those reported in adult studies and none of these medications were efficacious in promoting long-term smoking cessation among adolescent smokers. The decision to use pharmacotherapy in adolescents should be individualized and should be administered in addition to cognitive-behavioral counseling and support.

Nicotine dependence over time can result in neuro-plastic changes in the brain (163), and therefore there is a possible concern for nicotine replacement therapy use during adolescence, which is that nicotine can change the neurodevelopmental trajectory. Therefore, understanding how nicotine affects the adolescent brain, and identifying novel therapeutics is essential to treating nicotine addiction in adolescents.

Cessation interventions utilizing mobile devices and social media also show promise in boosting tobacco cessation. Technology-based smoking cessation interventions such as the tobacco quitting helpline and other telehealth approaches are not only cost effective but increase the likelihood of adults and adolescents quitting, compared with no intervention.

Pharmacogenomics approaches hold promise for personalized treatment by increasing success rates in nicotine dependence treatment (82, 164–172). Thus, effective treatments that support tobacco cessation in both adults and adolescents should include both behavioral therapies and FDA-approved medications and further emphasis be placed on personalization of cessation treatments to increase the possibility of compliance and ensure success of the intervention.

## Author Contributions

Manuscript was written by SM and reviewed extensively and conceptualized by GH and AQ. All authors contributed significantly to this article.

## Conflict of Interest

The authors declare that the research was conducted in the absence of any commercial or financial relationships that could be construed as a potential conflict of interest.
